# Examining Mobile Technologies to Support Older Adults With Dementia Through the Lens of Personhood and Human Needs: Scoping Review

**DOI:** 10.2196/15122

**Published:** 2019-11-11

**Authors:** Bon Mi Koo, Lisa M Vizer

**Affiliations:** 1 SSK Research Center for Mental Health and Communal Society Kwangwoon University Seoul Republic of Korea; 2 Division of General Medicine and Clinical Epidemiology School of Medicine University of North Carolina at Chapel Hill Chapel Hill, NC United States

**Keywords:** dementia, Alzheimer disease, mobile health, consumer health informatics, personhood, systematic review, smartphone, mobile phone, tablet computers

## Abstract

**Background:**

With the world’s rapidly growing older adult population, there is an increase in the number of people living with dementia. This growth leads to a strain on their caregivers and our health care system and to an increased attention on mitigating strain by using mobile technology to sustain the independence of people with dementia. However, less attention is given to whether these technologies meet the stated and unstated needs of people with dementia.

**Objective:**

The aim of this study was to provide an overview of the current research on mobile technologies for people with dementia, considering the current research through the lens of personhood and human needs, and to identify any gaps that represent research opportunities.

**Methods:**

We performed a systematic search in Medical Literature Analysis and Retrieval System Online (MEDLINE), Web of Science, PsycINFO, Cumulative Index of Nursing and Allied Health Literature (CINAHL), Excerpta Medica dataBASE (EMBASE), and the Cochrane Central Register of Controlled Trials (CENTRAL) in October 2018. We screened 5560 articles and identified 24 that met our inclusion and exclusion criteria. We then performed thematic analysis to organize the articles by the types of support mobile technologies provide and mapped those types of support to human needs to identify the gaps in support.

**Results:**

Articles described research on mobile technologies that support people with dementia to (1) perform daily activities, (2) maintain social interaction, (3) aid memory, (4) engage in leisure activities, (5) track location, and (6) monitor health. At least one type of support mapped to each human need, with most supporting lower-level needs such as physiological and safety needs. Little attention seems to be paid to personhood.

**Conclusions:**

Mobile technologies that support daily activities, relationships, memory, leisure activities, health, and safety can partially compensate for decreased function owing to dementia, but the human needs of people with dementia are often not adequately considered. Most technologies support basic physiological and safety needs, whereas many pay little attention to higher-level needs such as self-esteem and agency. Important research opportunities include using person-centered methods to develop technology to meet higher-level needs and to preserve personhood by incorporating human and psychological needs of people with dementia along with ethical considerations.

## Introduction

As the world’s population rapidly ages, the number of older adults with cognitive impairment will grow as well. Nearly 47 million people worldwide are now experiencing dementia, and this number is projected to triple by 2050 [1]. Of the diseases that can occur as people age, dementia is of particular concern to patients, their family and friends, and society as people with dementia progressively lose independence and autonomy, causing a sharp increase in the burden of care. In the early stages of dementia, people can benefit from support for complex tasks of daily living, but as the disease progresses, people become fully dependent on others to complete even basic activities of daily living [2,3].

Innovative technologies may assist people with dementia and informal carers (usually family or close friends, as opposed to health professionals such as nurses) in several ways. Assistive technology can decrease the burden of care [4-6], increase the independence of people with health conditions [7], and improve the well-being of people and their carers [8]. Advanced mobile technology designed for people with dementia, their carers, and even the surrounding environment has further expanded the scope of assistive technology available for people with dementia [9]. Examples [4-9] include partial compensation for functional deficits of people with dementia and support for carer’s care routines. Furthermore, ambient intelligence technologies embedded in the environment can increase safety and security by monitoring people with dementia in their homes to detect emergencies [10]. To date, however, little research has evaluated how mobile technologies specifically support daily activities of people with dementia.

Previous work [11-13] examining the role of human needs in the quality of life of people with dementia emphasizes the importance of considering the technologies designed for people with dementia in light of human needs as described by Maslow [14] and Kitwood [15]. Dewing’s [16] work also contributes context with his definition of personhood as “the attributes possessed by human beings that make them a person.” This definition is particularly applicable to people with dementia as it is inclusive and, unlike some other definitions of personhood, does not require that a person possess certain capabilities. Rather, it values any person’s possession of any attributes of humanity [16,17]. However, we found no research examining the role of these technologies fulfilling human needs and preserving personhood.

To address this gap, we reviewed the literature on mobile technologies intended for use by people with dementia in their daily lives and considered the current research through the lens of human needs and personhood. Our review defined 6 types of support that mobile technologies provide to people with dementia and caregivers, mapped the types of technology support to the human needs defined by Maslow [14] and Kitwood [15], and discussed considerations relating to technology adoption and ethics involved in developing technology for people with dementia.

## Methods

### Overview

We conducted a scoping review based on the methodological framework suggested by Arksy and O’Malley [18] and Peters et al [19], with the goal of organizing the literature on mobile technologies to assist people with dementia with daily living. We aimed to rapidly review the extent and range of research activity and map the key concepts underpinning the research area of mobile technology support for people with dementia [18]. The methodological framework of our scoping review includes identifying data sources and search strategies, selecting relevant articles, and extracting and charting the results.

### Data Source and Search Strategies

We performed a systematic search in October 2018 for articles published in Medical Literature Analysis and Retrieval System Online (MEDLINE), Web of Science, PsycINFO, Cumulative Index of Nursing and Allied Health Literature (CINAHL), Excerpta Medica dataBASE (EMBASE), and the Cochrane Central Register of Controlled Trials (CENTRAL). Systematic search terms included mobile devices, mobile app, smartphone, tablet, mild cognitive impairment, Alzheimer, dementia, and older adults. Multimedia Appendix 1 lists search strings per database.

### Study Selection

We selected articles based on the following inclusion criteria: (1) the article is published in a peer-reviewed journal in English, with full text available, (2) the article describes the study participants or target users as people with dementia aged 50 years or above and their caregivers, (3) the article describes mobile technology or mobile apps used by people with dementia and their caregivers to support daily activities of people with dementia, and (4) the article describes technology or apps intended for use outside of the clinic. We included study protocol or system design articles meeting our user criteria so that the review reflects the newest research trends. We focused on systems that used only smartphones and tablets as they are widely used, and these systems do not require purchase of specialized equipment. We excluded articles describing mobile technology to support people with illnesses other than dementia.

### Procedure

**Figure 1:**
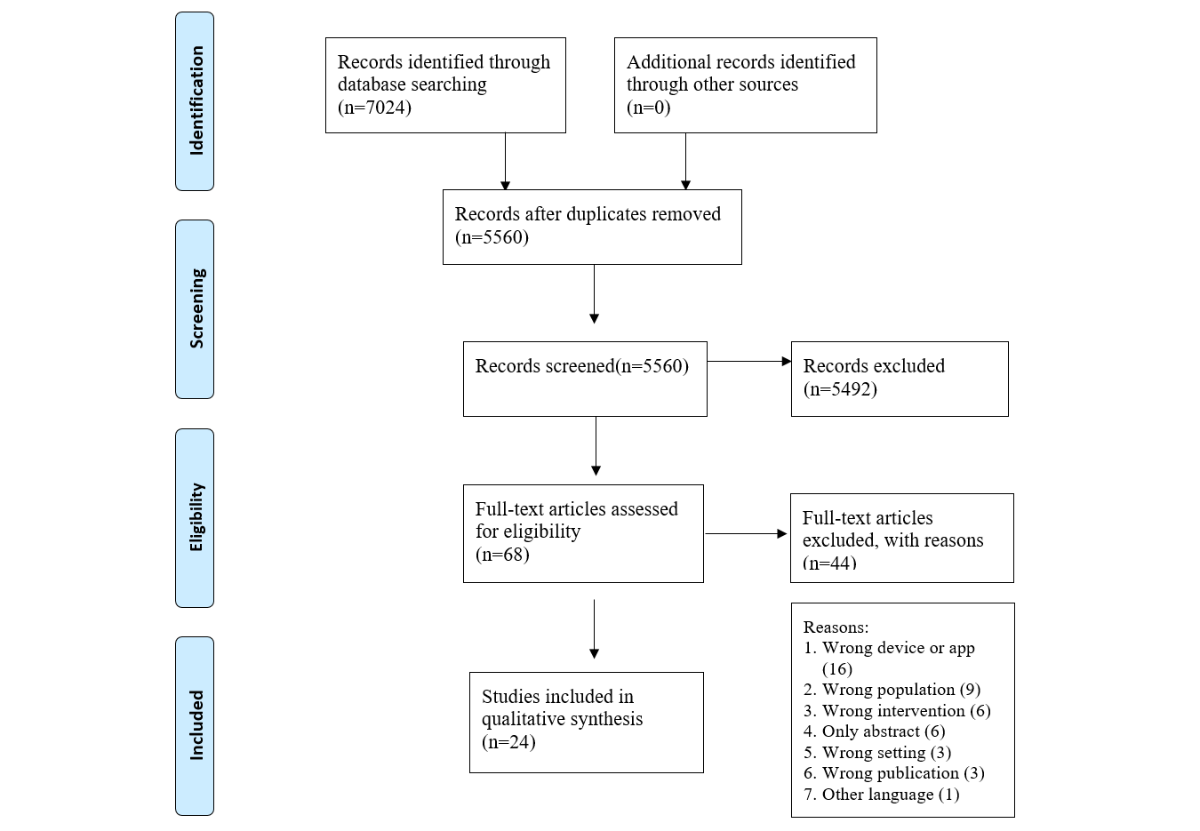
Preferred Reporting Items for Systematic Reviews and Meta-Analyses diagram.

We obtained 7024 articles from our initial search: 1132 from MEDLINE, 1760 from the Web of Science, 646 from PsycINFO, 211 from CINAHL, 3255 from EMBASE, and 20 from CENTRAL. We removed 1464 duplicate papers, then both authors independently performed title and abstract screening. We reconciled discrepancies in these screening results through consensus and then independently conducted full-text screening on 68 papers. Differences in the full-text screening results were also resolved through consensus. Finally, 24 papers met our inclusion criteria. The flow diagram of the search procedure is presented in Figure 1.

### Extraction and Charting of Results

After selecting the relevant articles, we conducted thematic analysis and summarized key information [19]. One researcher (BMK) undertook initial reading and used an inductive thematic analysis procedure to identify emergent themes related to the types of support the technologies provide and coded articles by the strategies used to support people with dementia. As coding progressed, new themes were incorporated, and previously coded studies were revisited. A second researcher (LMV) validated the themes after they were finalized. Discrepancies were discussed and resolved. To characterize the needs these systems can fulfill and the dignity they might afford people with dementia, we then matched the types of technology support identified in the thematic analysis to human needs using a mapping adapted from Barker and Board [20], and applied Dewing’s definition of personhood [14-16,21-23].

## Results

First, we show the year-to-year trends in publication volume and the types of technologies used for research on mobile technologies to support daily living for people with dementia. We then describe the types of support offered by the technologies in each article. Finally, we map those types of support to human needs to identify research opportunities.

### Publications per Year and Types of Technology

**Figure 2:**
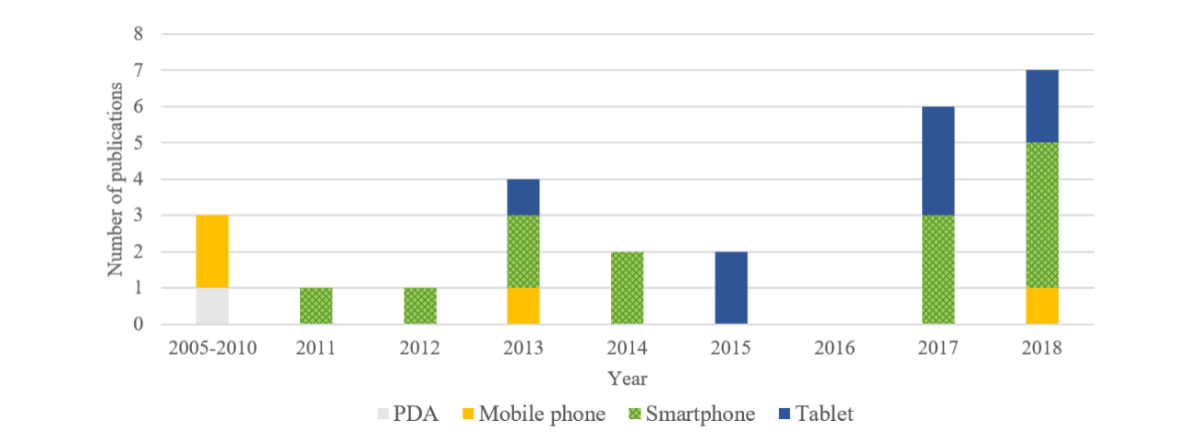
The number of publications per year (N=26). Publications by Lancioni et al [28] and Megges et al [38] are depicted twice as both a tablet and a smartphone were used in the study. We designated “mobile phone” when the article mentioned the use of a mobile phone without the use of apps or functions of a smartphone. PDA: personal digital assistant.

Figure 2 presents the number of publications per year, showing the increasing interest in this topic and the evolution of the types of devices studied over time. Our search produced only 3 articles published before 2010 that used mobile technology to support daily activities of people with dementia. The number of publications grew marginally between 2011 and 2015 but increased substantially in 2017 and 2018.

Recent articles show a preference for using smartphones and tablets to assist people with dementia with daily activities, reflecting the relative popularity of these mobile devices. Of the 24 studies, 17 used smartphone (n=13) and mobile phone (n=4) technologies, about twice the number describing tablets (n=8). About 71% (n=17) of the articles focused on the use of small devices.

### Mobile Technology to Support Independence and Well-Being of People With Dementia

Our review produced 24 articles meeting the inclusion criteria. We categorized those articles into 6 types of support that the technologies provided to people with dementia and caregivers. A total of 9 articles described technologies that support the performance of daily activities; 2 articles discussed technologies that facilitate social interactions; 1 article defined technology to aid retrospective memory; 2 articles examined technologies for supporting leisure activities; and 7 articles studied mobile technology for tracking location. Finally, 3 articles described technologies for health monitoring.

The next sections detail the technologies outlined in each article per category. Table 1 organizes the articles in the review by the type of support provided and whether they described developed systems or proposed systems. Table 2 provides a summary of each article organized by type of support, listing the system technologies and functions.

**Table 1:** Categorization of the articles reviewed.

Type of support		Tested systems		Proposed systems^a^		
		Tablet/PDA^b^	Smartphone/mobile phone		Tablet	Smartphone
**Performing daily activities (n=9)**						
	Providing reminders	Imbeault et al [24]	El Haj et al [25], Imbeault et al [26], and Imbeault et al [27]		—^c^	—
	Storing important information	—	Imbeault et al [26] and Imbeault et al [27]		—	—
	Providing sequential instructions for daily activities	Lancioni et al [28]^d^	Lancioni et al [28]^d^ and Lancioni et al [29]		Mahoney et al [30]	—
	Providing sequential instruction for way finding	Chang et al [31]	Kwan et al [32]		—	—
Maintaining social interaction (n=2)		Ekström et al [33]	—		—	Fardoun et al [34]
Aiding autobiographical memory (n=1)		—	De Leo et al [35]		—	—
Engaging in leisure activities (n=2)		Tyack et al [36] and Lim et al [37]	—		—	—
Tracking location (n=7)		Megges et al [38]^c^	Megges et al [38]^c^, Faucounau et al [39], Miskelly [40], and Olsson et al [41]		—	Ko et al [42], Solanas et al [43], and Xiao et al [44]
Monitoring health (n=3)		—	Zylstra et al [45] and Kamil et al [46]		—	Lin et al [47]

^a^Proposed systems: Article only described a prototype or a feasibility test with healthy users.

^b^PDA: personal digital assistant.

^c^Not applicable.

^d^These studies tested both smartphones and tablets, so they are listed twice.

**Table 2:** Summary of the articles reviewed.

Type of support and article		Technology	System functions	Study outcome
**Performing daily activities**				
	Chang et al [31]	PDA^a^, RFID^b^ tag, readers, and a routing engine. the server	Provides navigation guidance when the PDA is close to RFID tags on the wall at decision points inside a building	Using the navigation prompt system increased the correct performance rates of PwD^c^ on indoor wayfinding tasks
	El Haj et al [25]	Smartphone and Google Calendar	Sends 5 alerts for each of the targeted prospective events	Providing reminders improved task performance for individual PwD
	Imbeault et al [26] and [27]	Smartphone and AP@LZ app	AP@LZ supports daily activities such as recording a memo or setting reminders for scheduled activities	PwD successfully learned AP@LZ functions and used them for daily activities
	Imbeault et al [24]	Tablet and calendar app	A calendar app schedules appointments and activities	PwD successfully learned to use the calendar app to add notes, appointments, or activities to calendar; set alarms; attend appointments; and log past events
	Kwan et al [32]	Smartphone and a Map app	Sends verbal instructions from the Map to guide users to a destination	Study of using the smartphone app for navigation assistance demonstrated similar rates of acceptability and feasibility for groups with and without mild dementia
	Lancioni et al [29]	Tablet, the Talking Alarm Clock app, and a wireless Bluetooth earpiece	Sends reminders at predetermined schedules, verbal instructions, and brief encouragement	PwD using app showed higher rates of independently starting scheduled activities and performance accuracy
	Lancioni et al [28]	Smartphone or tablet, the Talking Alarm Clock app, and a wireless Bluetooth earpiece	Sends reminders at predetermined schedules, verbal instructions, and brief encouragement	PwD using app showed higher rates of independently starting and completing activities
	Mahoney et al [30]^d^	Tablet, Microsoft Kinect, fiducial tracking system, and RFID tags	Designed to identify dressing actions and clothing items, then provide guidance using sensors	N/A^e^
**Maintaining social interaction**				
	Ekström et al [33]	Tablet and the GoTalk NOW app	Creates a personalized communication book using GoTalk NOW and supports communication with caregiver using this app	Helped PwD and caregivers find conversation topics and helped PwD initiate communication
	Fardoun et al [34]^d^	Smartphone, smartwatch, and cloud architecture (server and database)	Designed to perform face recognition on a picture taken with a smartwatch and to return information about the person in the picture	N/A
**Aiding** **a** **utobiographical memory**				
	De Leo et al [35]	Smartphone, app, and server	Automatically takes pictures at 5-min intervals from 8 am to 8 pm, uploads pictures to a server overnight, and prepares a DVD with picture slides for delivery to the PwD	PwD demonstrated a higher correct recall rate for recent events
**Engaging in leisure activities**				
	Tyack et al [36]	Tablet and the art-viewing app	Art app using tablets loaded with photos of art objects, photographs, and paintings from 3 London museums	PwD showed improved well-being score at the end of art-viewing session
	Lim et al [37]	Tablet and 11 preselected apps	Uses 11 commercial leisure activity apps for creativity, simple games, and relaxation	Usable by those with little technology experience after training; half of the participants independently used apps for an average of 24 min per day
**Tracking location**				
	Faucounau et al [39]	Mobile device (GPS)	Sends regular monitoring messages to a caregiver’s phone, as well as alarms when it detects activity out of a preset safety zone, long periods of inactivity, or a fall	PwD and spouse reported issues with large device size, malfunctions, and usage difficulties after testing
	Ko et al [42]^d^	Smartphone and wandering path tracking and fall detection system	Designed to automatically take pictures and send them to a cloud system labeled with location and time	N/A
	Miskelly et al [40]	Mobile phone (GPS)	Regularly sends geographical position of the mobile phone to a central server for tracking	Tracking was 90% accurate but study had a high rate of noncompliance owing to comfort and usability issues
	Olsson et al [41]	Mobile phone (GPS) and the passive positioning alarm package	Sends a short message service text message with a map to the caregiver when the PwD is out of the safe zone	PwD and spouses developed trust in the alarm system over time, contributing to perception of value
	Megges et al [38]	Smartphone (GPS), tablet, and the app	Sends alarm and location information to the caregiver when PwD goes out of safe zone	Good initial usability and function ratings, but usability rating decreased after 4 weeks; however, most caregivers were willing to purchase the system
	Solanas et al [43]^d^	Smartphone (GPS) and m-Carer app	m-Carer app will send GPS data to a location server linked to a preference server with personal information	N/A
	Xiao et al [44]^d^	Smartphone with GPS, compass, and camera with fish-eye lens	Designed to send real-time snapshots, maps, and street views to the caregiver	N/A
**Monitoring health**				
	Zylstra et al [45]	Smartwatch and smartphone	Measures daily step count and maximum distance traveled from home	Trajectory of activity and GPS data provided a good estimation of functional status
	Kamil et al [46]	Cell phone (text messaging)	Collects fall reports via text message	Data from text messages were more accurate than data from calendars
	Lin et al [47]^d^	Smartphone and wandering behavior detection system: Outdoor Aider for Elders with Dementia	Designed to detect spatially disoriented behaviors by sending ongoing GPS trace to a server to analyze for pacing and lapping patterns	N/A

^a^PDA: personal digital assistant.

^b^RFID: radio-frequency identification.

^c^PwD: people with dementia.

^d^Proposed systems: article only described a prototype or a feasibility test with healthy users.

^e^N/A: not applicable.

#### Support for Performing Daily Activities

People with dementia often have difficulty performing daily activities owing to loss of memory and executive function [48]. Mobile technologies have been used as an external memory aid to help people with dementia remember future activities [25,26] and to provide visual and/or audio instructions for complex sequential tasks [29]. For step-by-step instructions, a context-aware system was used to detect situations or behavior through sensors in mobile devices [30,31] or clothing [30]. The portability of mobile devices and the use of a wireless Bluetooth earphone allowed people with dementia to be more aware of reminders and instructions in any location [28,29].

#### Provide Reminders for Upcoming Events

Reminder systems send alarms to help people with dementia to remember future events. In a case study [25], a person with mild Alzheimer disease used Google Calendar to remind her to perform scheduled activities without any help from her caregiver. In the intervention stage, the person with dementia received 5 smartphone alerts for each of the 3 targeted events: medical appointments, community club activities, and weekly church services. The study participant successfully performed the tasks supplemented with reminders more often than tasks without any reminders.

Imbeault et al [24] also studied the use of a commercially available calendar app on a tablet with people with dementia. The study showed that through repeated step-by-step instructions and training, a person with dementia could add notes, appointments, or activities to the calendar; set an alarm for upcoming events; and then successfully attend scheduled appointments. The participant also used the calendar app as a logbook for remembering past events, thus demonstrating the possibility of compensating for the loss of retrospective memory as well as prospective memory.

AP@LZ [26,27] is a multifunction smartphone app that provides a unique user interface specifically designed for people with dementia. This app includes reminders and other functions such as setting alarms, scheduling appointments, placing calls, and writing notes. A case study showed that participants with Alzheimer Disease could learn AP@LZ and perform daily activities with the help of reminders from AP@LZ. For example, participants used AP@LZ to set alarms to wake up, take medication, carry out activities, attend appointments, and lock the door at night.

#### Store Important Information for Future Use

People with dementia often experience difficulty remembering lists and important personal information, and mobile devices provide an easily accessible means of storing that information. For example, a person with dementia can keep a grocery list, maintain a medication history, or record personal information, such as name, age, address, phone number, and family contacts, for use in an emergency [26,27].

#### Provide Sequential Instructions for Daily Activities

The Talking Alarm Clock developed by Lancioni et al [28,29] supports people with dementia performing daily activities using reminders, step-by-step verbal instructions, encouragement, and praise. The system comprises a tablet device, the Talking Alarm Clock app, and a wireless Bluetooth earpiece linked to the tablet. Participants use the earpiece to hear alarms or instructions when they are not close to the tablet. The Talking Alarm Clock saves activity schedules with related instructions and praise statements. After an alarm and verbal reminder, specific instructions are provided to guide the person with dementia through each step. Some activities saved on the tablet include preparing coffee and breakfast, setting the table, watering plants, taking paper and soap to bathrooms, and making photocopies. Studies showed that this intervention helped people with dementia to start these activities by themselves and improved independent and accurate performance of these activities. However, the system could only provide prepared instructions and therefore could not alter instructions based on users’ behaviors.

Mahoney et al [30] developed a prototype of a complex system that detects users’ behaviors in context and provides accurate feedback to help people with dementia dress by themselves. The Development of Responsive Emotive Sensing System (DRESS) provided dressing guidance and track the sequence of dressing. The DRESS prototype comprised 4 parts: (1) a tablet (iPad) on top of the dresser to provides written, audio, or visual dressing guidance, (2) a Microsoft Kinect to track movements for dressing, (3) a fiducial tracking system to ensure that clothing is worn correctly, and (4) radio-frequency identification (RFID) tags on clothes and drawers to identify the clothing items that are removed. These elements allowed DRESS to detect correct and incorrect dressing actions and provide appropriate visual and audio guidance to people with dementia.

#### Provide Sequential Instructions for Way Finding

People with dementia often have difficulty navigating to a destination, either indoors or outdoors. The Ubiquitous Service for Direction Guide is an indoor way finding system [31] comprising a Personal Digital Assistant (PDA), Radio-Frequency Identification (RFID) tags, RFID readers, and a routing engine. For the study, RFID tags were installed at every decision point, such as doorways, corners, elevators, exits, or intersections of hallways, in a building. When the PDA is placed near a tag on the wall at a decision point, the reader interacts with the tag and presents a directional sign on the screen to aid navigation.

Kwan et al [32] developed an outdoor way finding system to help people with dementia with navigation. The system used GPS and a map app (Maps) installed on a smartphone to provide visual and verbal instructions to direct the person with dementia to a destination. To use the system, participants pressed the home button and initiated a voice command app to activate the Maps. They interacted with the Maps using earphones and a microphone. The study demonstrated that people with dementia could successfully navigate with assistance from a smartphone.

#### Support for Maintaining Social Interaction

People with dementia can have difficulty remembering or recognizing others’ names and faces, which can lead to social isolation. Mobile technology can facilitate social interaction by suggesting interesting conversational topics [33] or providing the persons with dementia with information about familiar people that they cannot remember [34].

The GoTalk NOW app [33] is a personalized communication book created by people with dementia and a trained specialist based on the person’s personal interests and activities, communication difficulties, and needs. In the case study, the specialist took pictures of objects and places that are meaningful to the subject and video-recorded activities that are familiar to her. Main topics are displayed on a home page, and link to other pages with relevant materials. Compared with traditional reminiscence materials such as scrapbooks and picture albums, the communication app can provide easy, organized access to more information and multimedia functions, making it more interactive and enjoyable. This study showed that the personalized communication app helped the person with dementia and her caregivers find conversation topics and helped them initiate communication.

Fardoun et al [34] described a system that uses a smartwatch, a smartphone, and cloud support to implement a face-detection system intended to help people with dementia recognize familiar people. To use the system, a person would take a picture of a person with a smartwatch; the watch sends the picture to a smartphone, and the phone uploads it to the cloud infrastructure. Face recognition is performed using a database and relevant information is delivered to the smartphone, which sends it to the smartwatch. The authors note their concern that people with dementia would have difficulty using the smartwatch’s small form factor.

#### Support for Aiding Autobiographical Memory

For people with dementia who have trouble remembering recent events, researchers are using mobile technology to aid autobiographical memory by providing a summary of daily events. De Leo et al [35] tested a system that used pictures to help people with dementia remember everyday events. Participants wore a smartphone around their necks on a lanyard and the smartphone took pictures every 5 min during the day. The images were transferred to a secure server and saved as slideshows labeled with date and time, and DVDs of the slideshows were mailed to participants once a week. Despite the relatively infrequent mailing, the study showed that watching slideshows helped people with dementia remember recent events. The authors demonstrated the feasibility of using a smartphone to augment retrospective memory for people with dementia by capturing, storing, and viewing pictures.

#### Support for Engaging in Leisure Activities

Mobile devices can deliver leisure activities for people with dementia with the advantages of intuitive interfaces, multimedia functions, and a wide variety of apps. People with dementia may be able to engage with these technologies independently, which can also benefit caregivers.

An art-viewing app developed by Tyack et al [36] enables people to enjoy art at home. The app shows pictures of objects, paintings, and photographs from several museums, collectors, and artists. Researchers tested the app with people with dementia and adjusted the interface, color, and font size to ensure easy manipulation of this app. The art-based intervention via a tablet increased interaction between people with dementia and other people, or between people with dementia and technologies, and encouraged people with dementia and their caregivers to create new activities that they could enjoy together.

Lim et al [37] studied several commercial apps to investigate whether people with dementia were able to use a tablet independently during leisure time. The apps encouraged people with dementia to play musical instruments, draw pictures, play games, and listen to music on tablets. The study found that even participants with little computer and technology experience could use the tablet after training sessions, and half of the participants independently used the apps during leisure time. Notably, 48% of the participants with dementia spent an average of 24 min per day using the tablet without supervision, and their caregivers expressed an intention to keep using the tablets on a regular basis.

#### Support for Tracking Location

Wandering is one of the more worrisome behaviors that people with dementia exhibit [39]. Wandering behavior increases the burden on family caregivers to keep the person with dementia safe, which can compel them to consider institutional care [39,41-43]. Mobile technology can help address this issue by providing detailed location information using GPS or photo and by providing alerts when movement is sensed outside a preset *digital fence*. Both of these approaches provide peace of mind and help caregivers find a lost family member as soon as possible. Among the 7 studies using mobile technologies for tracking location [38-44], 3 articles described new tracking systems that had not been tested with people with dementia [42-44].

For location tracking, systems usually require people with dementia to carry a GPS-integrated mobile device in a pocket [41,43], shoulder or waist bag [40], or waist belt [38,39] for continuous monitoring. Several studies described using a registered mobile phone to send geographical information to a server at predetermined time intervals for tracking and alerting caregivers based on predefined parameters [38-41]. GPS-integrated mobile phones can also be used to define a safety zone. If a person moves outside of that zone, an SMS message with location information and a map would be sent to the caregiver’s phone [38,39,41]. Considering privacy issues with continuous monitoring of people with dementia, Solanas et al [43] described the m-Carer app. It is designed to enable private monitoring by encrypting the information it sends to servers. The m-Carer app would regularly send encrypted data to the location server if the person with dementia stayed within a predefined area. In emergencies, however, it would send unencrypted location information to the server, which was forwarded to caregivers.

Some approaches use mobile devices to take pictures when the person with dementia is outside, requiring the smartphone to be worn on the chest, carried in a front pocket, or attached at the waist [42,44]. Ko et al [42] proposed the wandering path tracking and fall detection system (PTFaD), which takes pictures and sends real-time images, GPS location, and time to a server. This information is saved in the cloud and can be downloaded when necessary. In addition, the proposed PTFaD includes a fall detection system that measures the current direction of the smartphone using triaxial accelerometers. Xiao et al [44] described the Canderoid system to monitor the movement of people with dementia using multiple built-in smartphone sensors such as a GPS, compass, and camera. The proposed system uses a fish-eye lens for a wide-angle view, a digital compass for orientation, and GPS for location. This system would also give caregivers remote access to the smartphone, so they could use lasers to guide the subject back home.

#### Support for Monitoring Health

People with dementia can have difficulty expressing discomfort, symptoms, and unmet needs, so others may find it difficult to detect functional decline and provide assistance. However, mobile devices can be used to monitor functional status, predict functional decline, and detect neuropsychological behavior symptoms.

Zylstra et al [45] employed smartwatches and smartphones to monitor the functional status of people with dementia using daily step counts and the maximum distance that the study participants move from home. The smartwatch transfers activity and GPS data to the smartphone daily, which then uploads that information to a server. The study found that the trajectory of these data provides a good estimation of the subject’s functional status.

To track falls, a person with dementia or caregiver can record falls on a calendar, but this method suffers from recall bias and missing data. Kamil et al [46] addressed these issues by collecting fall data via text messaging. A secure platform sends a daily text message asking if a fall incident occurred the day before and records a *Yes* or *No* response from the person with dementia or caregiver. The authors showed that this method was more accurate than calendar data.

Lin et al [47] developed the Outdoor Aider for Elders with Dementia to detect disorientation, falls, inactivity, and wandering. A smartphone with GPS sends mobility data to a server, which then analyzes that data for pacing and lapping movement patterns to identify wandering behaviors often exhibited by people with dementia. Testing the algorithm on prerecorded sample GPS data showed that it could detect spatially disoriented behaviors.

### Mobile Technology for People With Dementia Through the Lens of Personhood and Human Needs

Table 3 shows a mapping between the needs defined by Maslow [14] and Kitwood [15], adapted from Barker and Board [20], along with the relevant technology support the reviewed systems provide for each need.

We found that at least one system included in the review provided support for each need, and technologies tend to support more basic needs such as physiological and safety needs. Reminder systems and instructions satisfy physiological needs by assisting people with dementia with basic activities [28-30] and satisfy safety needs by helping people with dementia with tasks such as navigating to a destination [31,32] or taking medications [26,27]. These systems also support agency and, therefore, satisfy esteem and identity needs by helping carry out activities unassisted, and thus maintain independence [24,25].

Health monitoring meets physiological and safety needs by observing functional status [45,46] and alerting others to falls [46]. Location functions meet safety needs by allowing caregivers to remotely monitor a person’s movements [38-44]. 

**Table 3:** Mapping between needs defined by Maslow (M) and Kitwood (K) and types of technology support provided by systems in the review.

Human needs	Definition	Articles	Type of support
Physiological (M)	Basic bodily and health needs	Lancioni et al [28], Imbeault et al [24], El Haj et al [25], Imbeault et al [26], Imbeault et al [27], Lancioni et al [29], Mahoney et al [30], Zylstra et al [45], Kamil et al [46], and Lin et al [47]	Send reminders, provide instructions, and monitor health
Safety (M)	Need for security	Lancioni et al [28], Megges et al [38], Imbeault et al [24], El Haj et al [25], Imbeault et al [26], Imbeault et al [27], Lancioni et al [29], Mahoney et al [30], Chang et al [31], Kwan et al [32], Faucounau et al [39], Miskelly [40], Olsson et al [41], Ko et al [42], Solanas et al [43], Xiao et al [44], Zylstra et al [45], Kamil et al [46], and Lin et al [47]	Send reminders, store personal information, provide instructions, track location, and monitor health
Comfort (K)	Need for a feeling of well-being	Ekström et al [33], Fardoun et al [34], Tyack et al [36], and Lim et al [37]	Aid interpersonal communication, provide information about familiar people, and provide entertainment
Attachment (K)	Need for reliable relationships	Ekström et al [33] and Fardoun et al [34]	Aid interpersonal communication and provide information about familiar people
Love and belonging (M); Inclusion (K)	Need for feeling accepted and included	Ekström et al [33] and Fardoun et al [34]	Aid interpersonal communication and provide information about familiar people
Occupation (K)	Need for rest and activity	Ekström et al [33], Fardoun et al [34], Tyack et al [36], and Lim et al [37]	Provide instructions, aid interpersonal communication, provide information about familiar people, and provide entertainment
Esteem (M); Identity (K)	Need for self-worth and autonomy	Lancioni et al [28], Imbeault et al [24], El Haj et al [25], Lancioni et al [29], Ekström et al [33], Fardoun et al [34], De Leo et al [35], Tyack et al [36], and Lim et al [37]	Send reminders, provide instructions, provide information about recent events and activities, aid interpersonal communication, and provide entertainment
Self-actualization (M); Agency (K)	Need for personal growth and freedom	Ekström et al [33], Fardoun et al [34], Tyack et al [36], and Lim et al [37]	Aid interpersonal communication, provide information about familiar people, and provide entertainment

Technologies for maintaining social relationships and aiding autobiographical memory directly support all needs except physiological and safety, by facilitating communication and providing information about people or events [33-35]. Spending time alone to pursue enjoyable and stimulating activities can meet the needs for comfort, occupation, esteem, identity, self-actualization, and agency [36,37]. Applying the concept of personhood [16], our review also showed that mobile technologies can facilitate positive person work [15,22] such as play [36,37], celebration [28,29], collaboration [33], and facilitation [28-32] to satisfy the psychological needs of people with dementia.

## Discussion

This scoping review surveyed the literature on using mobile devices to increase the independence and well-being of people with dementia, and then mapped those technologies to human needs [14,15]. This section will discuss trends that emerged across articles, and then discuss the implications raised by considering the research through the lens of personhood and human needs.

### Emerging Trends in Mobile Technology to Support Independence and Well-Being of People With Dementia

We organized the literature by how the technologies compensate for the loss of function that people with dementia experience in daily living. The 6 categories that emerged are (1) performing daily activities, (2) maintaining social interaction, (3) supporting autobiographical memory, (4) engaging in leisure activities, (5) tracking location, and (6) monitoring health status. Most mobile technology support was implemented for smartphones or mobile phones, implying that researchers believe that these devices are more suitable for supporting daily routines than PDAs or tablets.

The technologies that aid people with dementia with performing daily activities by providing instructions or reminders are the most mature among those in this review. Overall, 8 of the 9 articles in this category described studies in which people with dementia successfully used the technology [24,25,27-29,31,32]. Although these systems are not particularly complicated, they could fill an important role needed to promote independence.

We reviewed 2 articles concerning technologies that facilitate social interaction. The first article [33] discussed a case study that demonstrated the technology’s ability to aid conversation between a person with dementia and caregiver dyad. The other article [34] described an untested prototype intended to prompt people with dementia with information about people they are having trouble recognizing. In the analysis of human needs, we saw that social interactions are involved in meeting most needs; thus, supporting communication has the potential to aid many aspects of a person’s life. The lack of research in this area and value of social connection suggest an important opportunity for research.

A single article [35] described an aid for autobiographical memory and used technology that is now outdated. However, the study demonstrated that the idea of using pictures to augment memory is feasible. As autobiographical memory is vital for higher needs such as esteem and identity, and its loss is a hallmark of dementia, further research in this area could be valuable.

Research shows that enjoyable activities enhance quality of life, can reduce some behavioral symptoms of dementia, and ease caregiver burden [49]. The 2 articles [36,37] that involved using mobile technologies for leisure activity also reported positive outcomes; one showed an increase in well-being scores, and the other showed an increase in independent activity, along with a reduction in caregiver burden. Additional research in this area has the potential to enhance quality of life for both people with dementia and their caregivers.

A total of 7 articles [38-44] involved tracking devices intended to improve safety and address wandering behaviors common in dementia. However, 3 articles [42-44] only discussed prototypes, and 3 [38-40] of the 4 studies suffered from significant usability issues. This type of technology also raises important ethical concerns. Although these technologies could afford meaningful benefits, researchers must address usability and ethical issues before they can achieve more widespread use.

Overall, 3 articles discussed technology for monitoring the health of people with dementia. One [47] described an untested prototype for detecting wandering behavior, and 2 [45,46] studied simple measures that nonetheless provided good estimates of functional health. The modest research in this area suggests an opportunity for improved measures and interactivity.

Finally, a valuable finding demonstrated in several articles is that, despite impairments, people with dementia can successfully and meaningfully use mobile devices on a daily basis [24-29,31-33,35-37]. This result confirms the value of continued research in this area.

We noted that researchers are studying passively and unobtrusively collected data for monitoring the health status of people with dementia. Daily activity level and life space show trends in physical status [45], and patterns of outdoor movement observed from GPS traces can suggest wandering behavior [47]. Although in early stages, these studies mirror the trend in digital phenotyping research that is demonstrating that passive data from mobile devices can provide indicators of function and enable early detection of problematic behavioral symptoms [50]. Goals of that work include lessening reliance on self-report and reducing burden on the health system, patients, and caregivers.

Our review included articles describing technology specifically for people with dementia, but these technologies could possibly generalize to support people experiencing similar functional limitations owing to other illnesses or disabilities such as stroke, mental illness, brain injury, and physical or sensory disability. Widely popular mobile devices offer portability, connectivity, apps, and sensors that can provide support similar to dedicated assistive devices but eliminate the burden of carrying a separate device at all times and reduce stigma associated with more visible assistive devices [51]. However, people with limited function have specific accessibility needs that must be considered during design, and further research is needed to test efficacy and usability in these other user populations.

### Mobile Technology Support Through the Lens of Human Needs

By examining the types of support provided by the technologies in this review in the context of the needs of people with dementia, we found that many systems supported basic safety and physiological needs but much fewer facilitated higher-level needs. This is probably a consequence of the relative ease of using technology for providing objective information, such as locations, reminders, and instructions, versus providing individual support for creativity and personal growth. Technology for supporting higher-level needs must integrate insights from medicine and psychology to allow personalization, esteem-oriented feedback, and creativity. Other reviews also observed this gap, as a review of intelligent assistive technologies noted too little focus on the needs of people with dementia [52] and a review of how technology can address unmet needs discussed how behavioral and psychological needs were largely ignored [53]**.**

### Technology Adoption Through the Lens of Human Needs

Much literature discusses low uptake of health-related technology among older adults [21,52]. To understand human needs as a contributing factor, Thielke et al [21] applied Maslow’s hierarchy of needs to show how health-related technologies may inadvertently undermine the user’s independence. This can occur when a person believes that her lower-level needs are met, either as she is cognitively healthy or is unaware of her actual need, and therefore sees using a technology aimed at meeting those needs as threatening her autonomy. For example, a person who thinks he needs no help remembering directions is unlikely to adopt a way-finding technology. Some articles have labeled a dementia patient’s refusal to adopt a technology as *curious* [54] or report other describing people with dementia as *lazy* or *stubborn* [55], but a person with dementia may simply be unable to perceive his actual needs. Considering this, people with dementia are probably more likely to use systems that address needs they consider most important and may reject support for needs they do not believe they have, especially if they lack insight into their impairments. However, they may adopt systems that provide that support in the course of meeting other priorities. We might imagine that the same person who rejects a way-finding app might eagerly accept a technology that satisfies his desire to engage in community arts activities, which happens to provide directions to those events.

Thielke et al [21] also emphasized the importance of considering *who* uses a technology and *whose* needs that technology meets. Technology developed for use by people with dementia may actually help caregivers most by providing them peace of mind or free time to engage in leisure activities. For example, technology that detects wandering behavior is used by people with dementia and intends to increase their safety. However, the solution alerts *others* to problematic behavior, serving to give caregivers peace of mind but doing nothing in the moment for the person with dementia’s peace of mind. A person with dementia may reject this technology as she does not perceive that it provides for her own needs, though the solution does ultimately increase safety. Although people with dementia may be persuaded to use technology to appease caregivers [55], technology meant for use by people with dementia may be more successful if they can clearly see that it provides them with direct benefits in addition to caregivers.

For examples of systems that meet lower-level needs while also facilitating higher-level needs, we can look to accessibility-related technology developed for people with other impairments. One example is Aira [56], a system that supports people who are visually impaired by providing assistance with visual tasks such as outdoor navigation and clothing selection. Such a system might meet the security needs of a person with dementia in the course of facilitating esteem and self-actualization through autonomy. For instance, a technology such as Aira could help a person with dementia independently explore a new neighborhood by supporting safety through basic navigation assistance while also supporting agency and personal growth by highlighting personalized points of interest and making customized restaurant recommendations.

### Ethics Through the Lens of Personhood and Human Needs

Although not often discussed in the literature, we wish to highlight the importance of ethical considerations when working with and developing technology for people with dementia. Personhood [16] engenders a fundamental respect for human life, and people with dementia are particularly vulnerable to experiencing loss of that respect owing to cognitive decline, communication difficulties, and reliance on others for basic activities. Technologies that help compensate for functional deficits can partially protect against loss of personhood and help meet fundamental human needs, thus aiding in preserving the vital senses of self and dignity. However, the ubiquity of these technologies means that they become intertwined with all dimensions of a person’s life, thus introducing ethical concerns. Ethics should be considered early in the design process, with technology carefully developed within an ethical framework that serves the needs and protects the personhood of people with dementia. Technologies that undermine the personhood of people with dementia are those that work against their needs, value the needs of others over their needs, or value the personhood of others over their personhood. Considerations for mitigating the loss of personhood and meeting the needs of people with dementia include employing user-centered or value-sensitive design, ensuring informed consent, protecting privacy, and safeguarding data [54].

Using the concept of personhood [16], Maslow’s hierarchy of human needs [14], and Kitwood’s psychological needs of people with dementia [15] as organizing principles reveals that the significance of the technologies in this review goes beyond the surface functions of simply providing reminders or instructions. They can support personal dignity, fulfill vital needs, and help a person with dementia maintain independence and autonomy. This insight reinforces that mobile technologies for people with dementia should satisfy essential needs in a manner that retains and enhances personhood. To meet this goal, designers must understand the users of their technology and develop functionality that matches their aspirations and abilities [21]. A deep appreciation of user characteristics involves employing user-centered design methods [57] with a diverse sample of people with dementia and caregivers. Using these methods can improve adoption of mobile technology by people with dementia and promote positive outcomes. Above all, we must remember that people with dementia are not passive recipients of support but are people who actively desire to enrich their lives [58]. With continued research, we can design mobile technologies that more effectively meet their needs.

### Limitations

Limitations of this study pertain to the method and the technologies reviewed. First, we chose the scoping review approach as the research on mobile technologies to support people with dementia is still emergent and much current research is not yet published. As such, we included all articles that met our criteria and did not judge quality or exclude research still in progress, as advocated in Khalil et al [59]. We also chose to include only full-text English-language articles published in peer-reviewed journals or conference proceedings and may not have captured some pertinent research. These 2 factors combined account somewhat for the low number of included articles and limit our ability to assess the impact of these technologies and to make recommendations for practice. However, this method did allow us to illustrate the potential of and increased interest in using mobile technologies to support daily living and improve the independence and well-being of people with dementia and to identify opportunities for future research. We expect that the body of research will continue to evolve rapidly so that we can more systematically evaluate the efficacy and impact of these technologies and distill best practices.

Next, we focused only on standard mobile devices as they are widely available and increasingly already in use by people who develop dementia. However, this choice left out research on specialized technology, such as wearable technology or sensors installed in the environment [60], and systems that could be adapted for mobile technology in the future, such as reminiscence therapy using a laptop computer [61].

### Conclusions

This review summarizes the current research on mobile technologies that support daily activities for people with dementia. Our thematic analysis found that the current research describes the use of mobile devices to provide support for (1) performing daily activities, (2) maintaining social interaction, (3) aiding memory, (4) engaging in leisure activities, (5) tracking location, and (6) monitoring health. Further characterization of the literature using personhood and human needs for orientation finds that most work focuses on supporting safety and physiological needs and exposes an opportunity for work to support higher-order needs involving belonging, self-esteem, identity, and self-actualization. As research in this area matures, we expect continued innovations in technologies that meet higher-level needs, incorporate ethical considerations, employ user-centered methods, and are tested with large, diverse participant samples. Most importantly, we find that, beyond their mere functionality, the potential value of mobile technologies that support people with dementia lies in their ability to provide for vital human and psychological needs and reinforce personhood.

## Appendix

Multimedia Appendix 1 Search strings per database.

## Abbreviations

CENTRAL: Cochrane Central Register of Controlled Trials
CINAHL: Cumulative Index of Nursing and Allied Health Literature
DRESS: Development of Responsive Emotive Sensing System
EMBASE: Excerpta Medica dataBASE
MEDLINE: Medical Literature Analysis and Retrieval System Online
PDA: personal digital assistant
PTFaD: wandering path tracking and fall detection system
PwD: people with dementia
RFID: radio-frequency identification
