# Supplementary material for: Examining Mobile Technologies to Support Older Adults With Dementia Through the Lens of Personhood and Human Needs: Scoping Review
Source: JMIR Mhealth Uhealth. 2019 Nov 11;7(11):e15122. doi: 10.2196/15122 (PMC6878101; doi:10.2196/15122)
Supplement: Multimedia Appendix 1 [file mhealth_v7i11e15122_app1.docx]

**Appendix 1. Search strings per database**

**MEDLINE (via PUBMED, 1946 to present), Total: 1,132 Results**

("Computers, Handheld"[MeSH] OR "Cell Phones"[MeSH] OR mobile applications[mh] OR "handheld Computers" OR "handheld computer" OR "Cell Phones" OR "cell phone" OR "handheld computers" OR mhealth[tw] OR android[tw] OR ipad* OR iphone* OR messaging OR "text message" OR "text messages" OR "mobile device" OR "mobile devices" OR mobile phone* OR "mobile app" OR "mobile apps" OR "mobile applications" OR pda[tiab] OR pdas[tiab] OR smart phone* OR smartphone* OR tablet* OR "cellular phone" OR "cellular phones" OR "mobile telephone" OR "mobile telephones" OR "mobile application") AND (Aged[mesh] OR aged OR elder* OR octogenarian* OR centenarian* OR old OR older OR "older adult" OR "older adults" OR geriatric* OR "senior citizen" OR "senior citizens" OR senior OR seniors) AND (Dementia* OR dementia[mesh] OR dement* OR "alzheimer disease" OR Alzheimer* OR senil*[tiab] OR alzheimer disease[mesh] OR cognition OR cognitive OR cognitive dysfunction[mesh])

**WEB OF SCIENCE, Total: 1,760 Results**

("handheld Computers" OR "Cell Phones" OR "cell phone" OR "handheld computer" OR "mobile applications" OR "mobile application" OR mhealth OR android OR ipad* OR iphone* OR messaging OR "text message" OR "text messages" OR "mobile device" OR "mobile devices" OR “mobile phone*” OR "mobile app" OR "mobile apps" OR "mobile applications" OR pda OR pdas OR "smart phone*" OR smartphone* OR tablet* OR "cellular phone" OR "cellular phones" OR "mobile telephone" OR "mobile telephones" OR "mobile application") AND (aged OR elder* OR octogenarian* OR centenarian* OR old OR older OR "older adult" OR "older adults" OR geriatric* OR "senior citizen" OR "senior citizens" OR senior OR seniors) AND (Dementia* OR dement* OR Alzheimer* OR senil* OR cognition OR cognitive)

**PsycInfo via EBSCO, Total: 646 Results**

("handheld Computers" OR "Cell Phones" OR "cell phone" OR "handheld computer" OR "mobile applications" OR "mobile application" OR mhealth OR android OR ipad* OR iphone* OR messaging OR "text message" OR "text messages" OR "mobile device" OR "mobile devices" OR “mobile phone*” OR "mobile app" OR "mobile apps" OR "mobile applications" OR pda OR pdas OR "smart phone*" OR smartphone* OR tablet* OR "cellular phone" OR "cellular phones" OR "mobile telephone" OR "mobile telephones" OR "mobile application") AND (aged OR elder* OR octogenarian* OR centenarian* OR old OR older OR "older adult" OR "older adults" OR geriatric* OR "senior citizen" OR "senior citizens" OR senior OR seniors) AND (Dementia* OR dement* OR Alzheimer* OR senil* OR cognition OR cognitive)

**CINAHL via EBSCO, Total: 211 Results**

("handheld Computers" OR "Cell Phones" OR "cell phone" OR "handheld computer" OR "mobile applications" OR "mobile application" OR mhealth OR android OR ipad* OR iphone* OR messaging OR "text message" OR "text messages" OR "mobile device" OR "mobile devices" OR “mobile phone*” OR "mobile app" OR "mobile apps" OR "mobile applications" OR pda OR pdas OR "smart phone*" OR smartphone* OR tablet* OR "cellular phone" OR "cellular phones" OR "mobile telephone" OR "mobile telephones" OR "mobile application") AND (aged OR elder* OR octogenarian* OR centenarian* OR old OR older OR "older adult" OR "older adults" OR geriatric* OR "senior citizen" OR "senior citizens" OR senior OR seniors) AND (Dementia* OR dement* OR Alzheimer* OR senil* OR cognition OR cognitive)

**EMBASE, Total: 3,255 Results**

("handheld Computers" OR "Cell Phones" OR "cell phone" OR "handheld computer" OR "mobile applications" OR "mobile application" OR mhealth OR android OR ipad* OR iphone* OR messaging OR "text message" OR "text messages" OR "mobile device" OR "mobile devices" OR “mobile phone*” OR "mobile app" OR "mobile apps" OR "mobile applications" OR pda OR pdas OR "smart phone*" OR smartphone* OR tablet* OR "cellular phone" OR "cellular phones" OR "mobile telephone" OR "mobile telephones" OR "mobile application") AND (aged OR elder* OR octogenarian* OR centenarian* OR old OR older OR "older adult" OR "older adults" OR geriatric* OR "senior citizen" OR "senior citizens" OR senior OR seniors) AND (Dementia* OR dement* OR Alzheimer* OR senil* OR cognition OR cognitive)

**Cochrane Central Register of Controlled Trials (CENTRAL) (via Cochrane Library, 1948 to present), Total: 20 Results**

("handheld Computers" OR "Cell Phones" OR "cell phone" OR "handheld computer" OR "mobile applications" OR "mobile application" OR mhealth OR android OR ipad* OR iphone* OR messaging OR "text message" OR "text messages" OR "mobile device" OR "mobile devices" OR “mobile phone*” OR "mobile app" OR "mobile apps" OR "mobile applications" OR pda OR pdas OR "smart phone*" OR smartphone* OR tablet* OR "cellular phone" OR "cellular phones" OR "mobile telephone" OR "mobile telephones" OR "mobile application") AND (aged OR elder* OR octogenarian* OR centenarian* OR old OR older OR "older adult" OR "older adults" OR geriatric* OR "senior citizen" OR "senior citizens" OR senior OR seniors) AND (Dementia* OR dement* OR Alzheimer* OR senil* OR cognition OR cognitive)
